# Age, sex and ethnicity changes in creatine kinase and sex- and ethnicity-specific reference intervals of creatine kinase

**DOI:** 10.1016/j.clinme.2026.100596

**Published:** 2026-05-15

**Authors:** Tejas Kalaria, Rousseau Gama, Emma Tuddenham, Catherine Collingwood, Hem Sapkota, Latika Gupta, Mariam El Hawli, Rachel L. Griffiths

**Affiliations:** a Clinical Biochemistry, Black Country Pathology Services, The Royal Wolverhampton NHS Trust, Wolverhampton, UK; b School of Medicine and Clinical Practice, University of Wolverhampton, Wolverhampton, UK; c Clinical Biochemistry, South West London Pathology, London, UK; d Clinical Biochemistry, Alder Hey Children’s NHS Foundation Trust, Liverpool, UK; e Department of Rheumatology, Royal Wolverhampton Hospitals NHS Trust, Wolverhampton, UK; f School of Infection, Inflammation and Immunology, College of Medicine and Health, University of Birmingham, Birmingham, UK; g Francis Crick Institute, London, UK; h Paediatrics, Sandwell & West Birmingham Hospital NHS Trust, West Bromwich, UK

**Keywords:** Creatine kinase, CK, Ethnicity, Sex, Age, Reference intervals

## Abstract

**Background:**

Creatine kinase (CK) reference intervals (RIs) derived from predominantly White cohorts do not account for well-documented ethnic differences in CK. This misclassification may lead to unnecessary investigations or inappropriate treatment. We aimed to characterise age-, sex- and ethnicity-related variation in CK and establish RIs for a multi-ethnic population.

**Methods:**

Deidentified CK results of primary care requests between 2008 and 2024 from a laboratory in the West Midlands (UK) were partitioned into six groups (male and female of White, Asian and Black ethnicities). Indirect RIs were derived from 58,096 individuals (33,879 female; 24,217 male) with the refineR algorithm. Derived RIs were verified in two independent datasets.

**Results:**

Median CK and upper reference limits (URLs) were highest in Black, intermediate in Asian and lowest in White groups. Female URLs for ≥13 years were 389 IU/L (Black), 188 IU/L (Asian) and 170 IU/L (White); male URLs were 757 IU/L, 357 IU/L and 314 IU/L, respectively. Applying the UK Pathology Harmony sex-specific RIs to all ethnicities classified 22.6–30.3% of results from Black individuals as high, versus 5.1–7.7% of White individuals. Using the new ethnicity-specific RIs reduced high-result rates in Black men and women. In individuals of mixed ethnicity, CK levels aligned more closely with the parent group with higher CK. After excluding outliers, the 97.5th percentile CK values for female and male children <13 years of Black ethnicities were 1.22 and 1.28 times higher, respectively, than those of White ethnicities.

**Conclusions:**

Adoption of CK RIs stratified by sex and ethnicity will allow more precise interpretation of the results, supporting equitable clinical decision-making and appropriate use of healthcare resources.

## Highlights

• First validated sex- and ethnicity-specific creatine kinase (CK) reference intervals for the UK derived by indirect method from 58,096 individuals across White, Asian and Black ethnic groups.
• Individuals of Black and Asian groups show higher CK URLs than White counterparts.
• Applying non-ethnic reference intervals misclassifies up to 30% of results from individuals of Black ethnicities as elevated.
• Ethnicity-specific reference intervals improve interpretation of CK results and support equitable clinical care.

## Graphical Abstract

**Figure:**
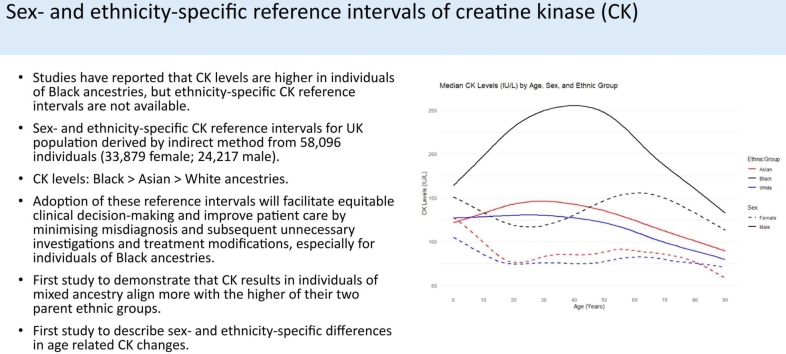

## Introduction

Creatine kinase (CK) is a non-specific marker of muscle damage and inflammation. It is widely used in clinical practice across paediatric and adult populations to investigate and monitor various metabolic and drug-induced myopathies. CK exhibits large inter-individual variation, with reference intervals confounded by various factors, including age, sex, body mass index and muscle mass.^1^, ^2^ Since the 1970s, studies have reported that baseline CK levels are significantly higher in individuals of African ancestry compared to those of European ancestry.^1^, ^2^, ^3^, ^4^, ^5^ Sex- and ethnicity-specific CK values from US population data of the National Health and Nutrition Examination Survey (NHANES) 2011–2014 are available as a guide to reference intervals;^2^ however, formal ethnicity-specific reference intervals are not available for use in routine practice. A UK harmonisation initiative (Pathology Harmony) recommended sex-specific CK reference intervals (males 40–320 IU/L, females 25–200 IU/L), with the caveat that these ranges were applicable for the White Caucasian cohort and that other ethnic groups may have higher values.^6^ No specific adjustments for different ethnicities were provided, and many laboratories still use reference intervals derived from older sources.^7^

CK is used for the diagnosis of muscular disorders as well as to guide treatment. For patients on statins, various guidelines recommend basing treatment initiation and/or continuation decisions on CK level relative to the upper limit of normal (ULN).^8^, ^9^, ^10^ Applying a single ULN for all ethnicities to the US NHANES data, CK was increased in 29.8% of Black men and 19.3% of Black women, compared to only 5.6% and 5.0% of White men and women, respectively.^2^ Median CK levels for the Black male population (214 IU/L) were shown to be higher than the stated assay ULN (200 IU/L), and a disproportionate number of Black men and women would have CK levels greater than three times the ULN compared to their White counterparts, with adjusted odds ratios of 5.97 and 1.98, respectively.^2^ These findings underscore the importance of applying CK reference intervals validated for the population served by the laboratory. The lack of ethnicity-adjusted reference ranges could lead to unnecessary referrals to secondary care for individuals belonging to ethnicities with physiologically higher CK levels, subjecting patients to avoidable anxiety and investigations, and substantial workload and cost implications for the health service. While studies have provided guide to sex- and ethnic-specific CK values for US-based populations,^2^ equivalent data for European cohorts have been lacking, and fully stratified CK reference intervals have not been established. This study, therefore, aimed to characterise age-, sex- and ethnicity-related variation in CK and to establish appropriate reference intervals stratified by ethnicity and sex for a diverse multi-ethnic UK population.

## Methods

In this registered and approved service improvement audit, deidentified results, age on the day of sample collection, sex and ethnicity were retrieved for all primary care CK requests from 1 January 2008 to 30 April 2024 from laboratory information management systems (LIMS) of laboratories serving a cosmopolitan population in the Black Country region of the West Midlands in England. For individuals with multiple requests within the period, only the first reported result was retrieved. The recorded ethnicity in the LIMS for primary care requests is the individual’s self-reported ethnicity in their primary care records as per the national ethnicity codes in England, which is retrieved at the time of electronic test request.^11^ Results from individuals whose ethnicity was not stated or was not available were not retrieved. The largest proportion of results originated from Sandwell and adjoining areas, and therefore, the dataset was compared with the 2021 Census ethnicity proportions in Sandwell for ethnicity representativeness in the dataset.

Most serum CK results during the data inclusion period were measured on Abbott Architect® c16000 (Abbott Diagnostics, IL, USA), whereas a few thousand CK towards the end of the inclusion duration were measured on Abbott Alinity C (Abbott Diagnostics, IL, USA) and Roche cobas® c 702 (Roche Diagnostics, Germany). The CK assays are harmonised to the IFCC (International Federation of Clinical Chemistry and Laboratory Medicine) reference measurement procedure,^12^, ^13^ there is no significant method-related difference (external quality assurance data) and, therefore, the results from both Abbott and Roche methods were included. However, this assumption was confirmed by assessing the difference between Abbott and Roche method CK results using multivariate regression. CK was modelled with a gamma generalised linear model with a log link to assess Abbott vs Roche differences when adjusted for age (modelled using a natural cubic spline with 4 degrees of freedom), sex and ethnicity.

Statistical analyses were performed using IBM SPSS Statistics for Windows, version 26 (IBM Corp.) and RStudio version 2024.12.0+467 for Windows using packages dplyr, tidyr, stats, multcomp, MASS, purr, splines and ggplot2. Kruskal–Wallis H test was used to compare differences in three or more groups and Dunn’s test with Bonferroni adjustment was used as a post-hoc test for pairwise comparison. Centile plots of CK changes with age were prepared using GAMLSS (Generalised Additive Models for Location, Scale and Shape) version 5.4-22 and ggplot2 in RStudio version 2024.12.0+467. P-splines using the singular value decomposition function (pb()) were used in the Box-Cox transformed distribution in the GAMLSS for the centile plots.^14^, ^15^

The reference intervals were derived using refineR, an algorithm for the derivation of reference intervals from a mixed distribution of real-world pathological and non-pathological test results, version 1.6.2 in RStudio version 2024.12.0+467.^16^ The refineR algorithm works on the assumption that the majority of results in the dataset are non-pathological, and these non-pathological results form a normal distribution when Box-Cox transformed using optimised parameters. The algorithm also assumes that the proportion of pathological results is insignificant in one region of the transformed distribution, and this is used to identify and separate pathological from non-pathological results in the whole distribution. The identified non-pathological distribution in the optimal model is then used to derive reference interval percentiles.^16^, ^17^ One thousand bootstrap iterations were performed for each partition. The median of the bootstrap percentile estimates for the 2.5th, 50th and 97.5th percentiles of the respective partition were reported as point estimates, along with their 95% confidence intervals. The 2.5th and 97.5th percentiles were reported as lower and upper reference intervals, respectively. Examples of the R codes used are in the Supplementary material.

For the purpose of creating ethnicity partitions for derivation of ethnicity- and sex-specific CK reference intervals, CK results from individuals ages 18 and above were compared for all White ethnicities (White – British, White – Irish, White – any other White background), Asian ethnicities (Asian or Asian British – Indian, Asian or Asian British – Pakistani, Asian or Asian British – Bangladeshi, other ethnic groups – Chinese) and Black ethnicities (Black or Black British – Caribbean, Black or Black British – African, Black or Black British – any other Black background) were compared. Results from individuals with mixed ethnicity (Mixed – White and Black Caribbean, Mixed – White and Black African, Mixed – White and Asian, Mixed – any other mixed background) were compared separately.

### Verification of derived reference intervals

Once derived, the reference intervals were applied to the dataset from which they were derived and two additional datasets to assess the proportion of results outside the reference intervals. The first additional dataset was primary care CK results between 1 May 2024 and 31 October 2024 from the Black Country Region of the West Midlands in England. The majority of the CK levels were measured by the Abbott Alinity C method, whereas a minority were measured by the Roche cobas e801 method at a total of six laboratory sites. The second independent dataset was primary care CK results between 26 January 2023 and 22 October 2024 from south-west London in England. The CK levels were measured by the Roche cobas method at a total of three laboratory sites.

## Results

CK results from a total of 59,644 individuals (34,733 female; 24,911 male) were included. CK results, ethnicity and sex descriptives of the included individuals are shown in Table 1. In the 2021 Census, 57.3%, 25.8%, 8.7% and 4.3% of individuals in Sandwell were White, Asian (including Asian British or Asian Welsh), Black (including Black British, Black Welsh, Caribbean or African) and mixed or multiple ethnic groups, respectively.^18^ In comparison, the retrieved dataset had results from 55.6%, 31.8%, 10.8% and 1.5% individuals of White, Asian, Black and mixed ethnicities, respectively.

**Table 1:** CK results, ethnicity and sex distribution of included individuals. CK results in IU/L are median with IQR in parentheses. n is the number of individuals in each group.

National ethnicity code	Ethnic group	Female		Male	
		n	CK (IU/L)	n	CK (IU/L)
A	White – British	16,590	78 (57–109)	12,339	109 (75–164)
B	White – Irish	255	78 (57–104)*	231	98 (68–157)*
C	White – any other White background	2,237	79 (58–114)*	1,485	112 (75–170)*
D	Mixed – White and Black Caribbean	364	116 (84–176)	190	208 (133–316)$
E	Mixed – White and Black African	87	107 (75–148)	52	213 (138–321)$
F	Mixed – White and Asian	117	90 (72–117)#	85	123 (90–188)#
G	Mixed – any other mixed background	0	-	0	-
H	Asian or Asian British – Indian	6,304	86 (64–120)	4,628	131 (91–198)
J	Asian or Asian British – Pakistani	3,009	84 (62–118)#	2,120	128 (89–190)#
K	Asian or Asian British – Bangladeshi	932	86 (63–118)#	640	134 (96–190)#
L	Asian or Asian British – any other Asian background	683	84 (63–114)#	626	135 (94–197)#
M	Black or Black British – Caribbean	2,526	151 (105–225)	1,521	222 (144–357)
N	Black or Black British – African	845	114 (82–172)	533	220 (139–356)$
P	Black or Black British – any other Black background	676	140 (96–210)$	362	225 (150–364)$
R	Other ethnic groups – Chinese	108	86 (67–118)#	99	132 (94–166)#
S	Other ethnic groups – any other ethnic group	0	-	0	-
Total		34,733		24,911	

* p > 0.05 compared to White – British of respective sex.

# p > 0.05 compared to Asian or Asian British – Indian of respective sex.

$ p > 0.05 compared to Black or Black British – Caribbean of respective sex.

CK results from 11,453 individuals from 29 September 2020 onwards included results from both Abbott and Roche methods. After adjustment for age, sex and ethnicity, there was no evidence of a difference in CK between the two included methods (Roche to Abbott results adjusted ratio 0.92, 95% CI 0.74–1.14, p = 0.414).

CK results for White ethnicities (White – British, White – Irish, White – any other White background) were similar and therefore treated as a single group for subsequent analysis. Similarly, CK results from Asian ethnicities (Asian or Asian British – Indian, Asian or Asian British – Pakistani, Asian or Asian British – Bangladeshi, other ethnic groups – Chinese) were similar and grouped, and CK results from Black ethnicities (Black or Black British – Caribbean, Black or Black British – African, Black or Black British – any other Black background) were grouped (Table 1). This created a total of six groups for subsequent analysis – male and female sex groups for each of White, Asian and Black ethnicities.

CK results from individuals with mixed ethnicities were closer to the ethnicity with higher CK levels out of the two parent ethnicities. For purity, the results from mixed ethnicities were kept separate. However, in view of the sample size limitation, CK reference intervals for mixed ethnicities could not be derived.

**Fig. 1:**
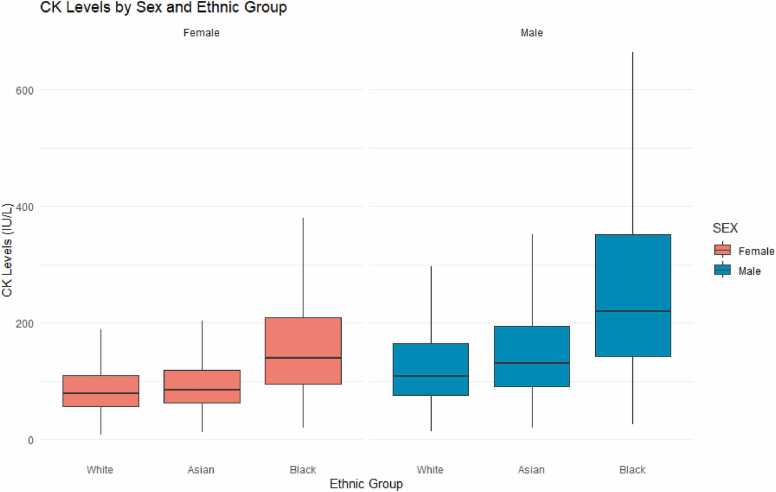
Box and whisker plots of CK distributions in women and men of White, Asian and Black ethnicities in individuals ≥13 years of age.

CK in women and men of Black ethnicities was higher compared to Asian and White ethnicities (all p < 0.001); and CK in women and men of Asian ethnicities was higher compared to White ethnicities (both p < 0.001, Fig. 1).

### Changes in serum CK levels with age

**Fig. 2:**
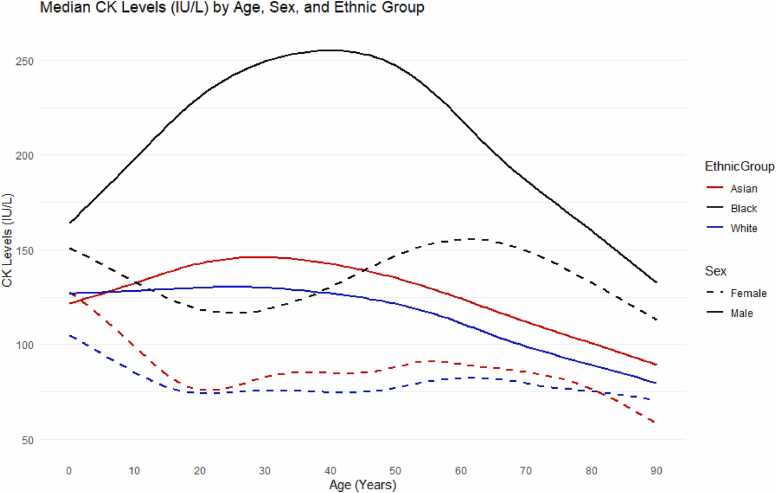
Median CK levels by age for sex and ethnicity groups.

Median CK levels with age for sex and ethnicity groups are presented in Fig. 2, and distributions of CK with age are in Supplementary Figure 1. CK levels in women gradually decreased during childhood until the age of 13 years, when they approximated adult levels. CK levels in women increased towards the end of the reproductive age (Black >> Asian > White) before slowly declining with advancing age. CK levels in men gradually increased during childhood (Black >> Asian > White) until the age of 13 years, when they approximated adult levels. CK levels in men peak between the ages of 20 and 50 years and thereafter gradually decrease with advancing age (Black >> Asian > White). CK levels and changes with age are much greater in female and male individuals of Black ethnicities, especially for Black men, compared to the other two ethnicities.

### Sex- and ethnicity-specific reference intervals of CK for individuals aged 13 years and older

The reference intervals were derived from a total of 58,096 individuals (33,879 female; 24,217 male). The refineR algorithm identified 92.5% to 96.3% of CK results from the six sex and ethnicity groups as results constituting non-pathological distribution, and the reference intervals were derived from these results. The algorithm output for individual sex and ethnicity groups is plotted in Supplementary Figure 2. The derived lower reference limit (LRL, 2.5th percentile), median and upper reference limit (URL, 97.5th percentile) are presented in Table 2.

**Table 2:** Sex- and ethnicity-specific serum CK reference intervals for individuals aged 13 years and older. UK Pathology Harmony CK reference intervals, for White Caucasians only, are included for comparison.^6^

Group	Number of individuals	CK lower reference limit^a^ (IU/L)	Median^a^ CK (IU/L)	CK upper reference limit^a^ (IU/L)
**Female**				
UK Pathology Harmony	-	25	-	200
White	18,978	29 (27–32)	75 (71–77)	170 (142–188)
Asian	10,894	35 (32–37)	82 (76–85)	188 (141–206)
Black	4,007	46 (41–49)	137 (133–140)	389 (345–422)
**Male**				
UK Pathology Harmony	-	40	-	320
White	13,927	35 (34–37)	106 (104–108)	314 (294–333)
Asian	7,927	44 (42–46)	125 (122–128)	357 (329–386)
Black	2,363	58 (50–64)	218 (210–227)	757 (660–842)

a The point estimates are the median of 1,000 bootstrap iterations and numbers in the brackets indicate 95% confidence intervals (CI).

For women and men, LRL and URL were much higher in Black ethnicities compared to Asian ethnicities, which in turn had slightly higher LRL and URL compared to White ethnicities. CK URL for women of Black ethnicities was 2.3 and 2.1 times higher than CK URL for women of White and Asian ethnicities, respectively. CK URL for males of Black ethnicities was 2.4 and 2.1 times higher than CK URL for males of White and Asian ethnicities, respectively. CK URLs for men and women of Asian ethnicities were 1.1 times higher than for White ethnicities.

### Sex- and ethnicity-specific reference intervals of CK for individuals aged under 13 years

**Fig. 3:**
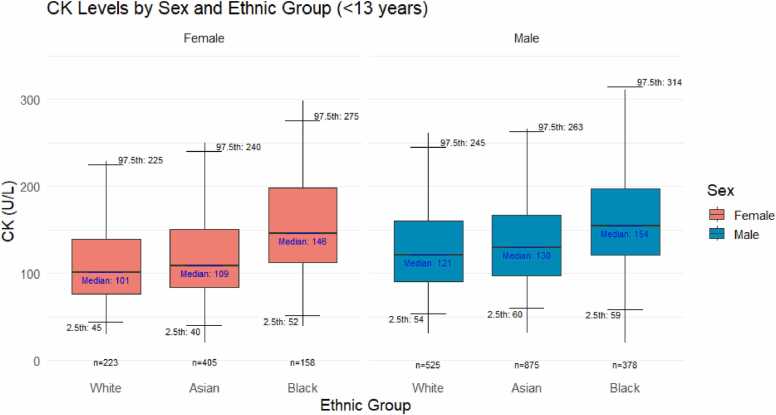
Creatine kinase (CK, U/L) in female and male children <13 years of White, Asian and Black ethnicities. Boxplots are plotted after removing outliers within each Sex×Ethnicity subgroup using Tukey’s rule (values <Q1−1.5×IQR or >Q3+1.5×IQR). Horizontal ticks mark the 2.5th and 97.5th percentiles of the trimmed distributions. Counts under the X-axis labels are post-exclusion numbers.

CK is not commonly requested for children in primary care and only 653 children under the age of 13 years were in the original dataset, both sexes and all ethnicities combined. CK, however, is often requested as part of initial screening blood tests for various conditions, for example, developmental delay, in community paediatrics clinics and in secondary care clinics. Therefore, for age under 13 years, a subsequent CK gather included all primary care, community paediatrics and secondary care outpatient CK results for dates 1 January 2008 to 22 October 2024 for both the included datasets. Despite that, the numbers were insufficient (a total of 2,464 results for all groups combined) to derive sex- and ethnicity-specific reference intervals for children under the age of 13 years. CK levels in female and male children under 13 years from Black ethnic backgrounds were significantly higher than those in children from Asian and White backgrounds (all p < 0.001). CK levels in female (p = 0.05) and male (p = 0.028) children under 13 years from an Asian ethnic background were slightly higher than those in children from a White ethnic background (Fig. 3). The 2.5th and 97.5th percentiles of the trimmed distribution (after outlier exclusion) shown in Fig. 3 are not reference intervals, but are to demonstrate differences between the sexes and ethnicities. Median CK for female and male children of Black ethnicities was 1.44 and 1.27 times higher than that of White ethnicities, respectively. The 97.5th percentile CK for female and male children of Black ethnicities was 1.22 and 1.28 times higher than that of White ethnicities, respectively. Although significant, ethnicity-related differences in CK levels among children under 13 years were smaller compared to those observed in individuals aged 13 years and older (Fig. 1, Table 2, Fig. 3).

### Sex- and ethnicity-specific reference intervals of CK for individuals of advanced age

Serum CK levels decrease with age for men and women of all three ethnic groups (Fig. 2, Supplementary Figure 1). The decrease is apparent after the age of 60 years and continues with further ageing. However, this is a gradual change and therefore will require multiple age partitions for all six groups to accommodate the transition. Limited sample size prevented the derivation of robust age-partitioned reference intervals for advanced ages for the six sex and ethnicity groups.

### Verification of derived reference intervals

When the derived sex- and ethnicity-specific reference intervals for individuals aged 13 years and over were applied to the same dataset from which they were derived, a different but related dataset (subsequent data from the same geography and laboratories) and an independent dataset (CK measured in unrelated laboratories in a distant geography from the same country – south-west London), the proportion of results outside the reference intervals was comparable for both the sexes of the three ethnicities. Depending on the dataset, sex and ethnicity group, 1.2–4.5% of results were less than the LRL and 5.7–10.2% of results were higher than the URL (Table 3).

**Table 3:** Assessment of derived sex- and ethnicity-specific CK reference intervals to compare the proportion of results outside the reference intervals for individuals aged 13 years and over in the dataset from which the reference intervals are derived and two additional datasets. For comparison, the proportion of results outside the reference interval is provided if the sex-specific UK Pathology Harmony CK reference intervals for White Caucasians are applied to all ethnicities.

Group	West Midlands 1 (dataset from which reference intervals are derived)				West Midlands 2 (subsequent data from the same geography and laboratories)			South-west London		
	n	% <LRL	% >URL	% >5×URL	n	% <LRL	% >URL	n	% <LRL	% >URL
**Applying sex- and ethnicity-specific reference intervals derived in this study**										
**Female**										
White	18,978	2.3%	8.0%	0.26%	1,229	3.1%	8.1%	709	2.7%	10.2%
Asian	10,894	2.7%	6.7%	0.19%	489	4.5%	7.4%	287	4.2%	7.3%
Black	4,007	2.4%	6.5%	0.35%	155	2.6%	9.7%	337	3.0%	6.2%
**Male**										
White	13,927	2.5%	6.0%	0.45%	805	3.7%	7.8%	511	1.2%	7.6%
Asian	7,927	2.3%	7.2%	0.63%	327	3.1%	6.7%	249	1.6%	8.0%
Black	2,363	1.6%	6.6%	0.68%	70	1.4%	5.7%	183	1.6%	7.1%
**If sex-specific but ethnicity non-specific UK Pathology Harmony reference intervals are to be applied to all ethnicities**										
**Female**										
White	18,982	1.2%	5.1%	0.25%	1,229	1.6%	5.7%	709	2.0%	6.2%
Asian	11,006	0.6%	5.7%	0.17%	489	1.2%	6.5%	287	2.1%	4.9%
Black	4,442	0.1%	28.1%	0.80%	155	0.7%	22.6%	337	0.3%	28.2%
**Male**										
White	13,927	3.9%	5.8%	0.45%	805	4.5%	7.7%	511	2.4%	7.4%
Asian	7,997	1.6%	9.0%	0.75%	327	2.1%	9.5%	249	1.6%	8.8%
Black	2,594	0.2%	30.3%	1.82%	70	0.0%	24.3%	183	0.6%	29.0%

If the UK Pathology Harmony sex-specific CK reference intervals, which are for White Caucasians only, are to be applied to all ethnicities, then women and men of Black ethnicities would have a negligible proportion (0.0% to 0.7%) of results less than the LRL, whereas 22.6% to 30.3% of results would be higher than the URL. Raised CK to a level >5 times the upper reference interval is used to make a decision on treatment modifications in certain scenarios, for examples for statin treatment.^8^ Respectively, 0.80% and 1.82% of women and men of Black ethnicities had CK results five times higher than the UK Pathology Harmony sex-specific URL, compared to 0.25% and 0.45% of women and men of White ethnicities (Table 3).

## Discussion

Previous studies in the USA and elsewhere have highlighted CK differences across demographic groups,^1^, ^2^ but no comprehensive reference interval data have been available for European populations. A comparable study in the Netherlands examined CK distributions in White Europeans and Surinamese immigrants (the latter of West African and South Asian ancestry), finding substantially higher CK values in the Surinamese subgroups and underscoring the need for population-specific reference ranges.^19^ Our work now provides such data for a contemporary multi-ethnic UK cohort.

A key strength of this study is the use of an indirect approach to derive reference intervals from routine laboratory data. Unlike traditional (‘direct’) methods that rely on small cohorts of healthy volunteers – who may not represent the population typically tested in clinical practice – indirectly established reference intervals better reflect the characteristics of the actual patient population undergoing routine testing (*normal vs reference*).^20^, ^21^ When multiple reference interval partitions are required, using the direct reference interval methods could be challenging because of ethical reasons, resource limitations, and statistical uncertainty from sampling bias.^16^, ^22^ Indirect methods, however, provide a pragmatic alternative in these scenarios by leveraging large datasets from routine practice, with additional advantage in the form of potential for being automated or semi-automated.^22^ Most indirect methods assume that results from healthy subjects follow a near-Gaussian distribution after transformation and this feature is used for separating pathological results from non-pathological results, before the reference intervals are then derived from the identified non-pathological results.^16^, ^23^ Many modern indirect reference interval methods achieve superior or comparable results to direct methods when the dataset is large and the proportion of pathological results is relatively small, generally <20%.^22^ By capturing real-world variability, our indirectly derived CK ranges should be immediately applicable to clinical practice.

Our findings indicate that applying the UK Pathology Harmony cut-offs without ethnic adjustment would lead to over-flagging of results in certain groups. Specifically, 22.6–30.3% of CK results from Black individuals in our cohorts would be classified as ʻhigh’ using the UK Pathology Harmony reference interval, compared to only 5.1–7.7% in White individuals. A higher-than-expected proportion of results from Asian individuals (5.7–9.5%) would also exceed the URL. This over-reporting of ʻabnormal’ CK results could trigger unnecessary repeat testing, specialist referrals, and further investigations. The National Institute for Health and Care Excellence (NICE, UK) clinical guideline for lipid management (NG238, 2023) states that statin therapy should not be initiated if there is a history of persistent unexplained muscle symptoms with CK >5×URL, and that a lower statin dose should be used if CK is elevated but <5×ULN.^8^ Using reference limits that are unsuitably low for certain ethnic groups may therefore lead clinicians to withhold or decrease the dose of statins inappropriately. In patients with muscle disorders such as myositis, applying inappropriately lower CK reference thresholds can lead to unnecessary treatment escalation, dose adjustments or even prolonged therapy, despite the disease being stable. In the absence of ethnicity-specific ranges, Black patients, particularly Black men, are most likely to be affected by such unintended consequences of over-investigation and avoidable treatments.

The magnitude of increased CK in Black and Asian ethnicities in this study is similar to that reported by George *et al* from the US NHANES analysis.^2^ The NHANES study compared 90th, 95th, and 97.5th percentiles of CK in different ethnicities rather than providing reference intervals after outlier exclusion. George *et al* found that 95th and 97.5th percentile CK was 2.3 and 2.6 times higher for Black men and 1.2 and 1.4 times higher for Asian men, respectively, compared to White men.^2^ In our study, the CK URL (97.5th percentile of outlier-excluded distribution) for Black men and Asian men were 2.4 and 1.1 times higher compared to White men, respectively. George *et al*. reported 95th and 97.5th percentile CK were 1.7 and 1.7 times higher for Black women compared to White women, whereas Asian women had slightly lower CK compared to White women (95th and 97.5th percentile 0.9 and 0.7 times, respectively).^2^ In contrast, we observed that the CK URL for both Black women and Asian women was 2.3 and 1.1 times higher compared to White women, respectively. These discrepancies may reflect differences in the ethnic subgroups or other population factors influencing CK levels in the cohorts.

One of the main limitations of ethnicity-specific reference ranges is that the definition of ethnic groups is not standardised internationally. Therefore, results from one population may not be directly generalisable to another. For example, ʻAsian’ in a US context predominantly includes individuals of Indian, Korean, Chinese, Vietnamese and Filipino descent, whereas in the UK ʻAsian’ ethnicity largely comprises individuals of South Asian descent (eg Indian, Pakistani, Bangladeshi). Similarly, ʻBlack’ in the USA typically denotes African-American individuals, while in the UK it commonly refers to Black African or Black Caribbean individuals. This lack of harmonisation in ethnic definitions means that studies like ours must be interpreted in the context of the local demographics and international comparisons of ethnicity-specific reference ranges may not be appropriate. A limitation of studies like this, reliant on ethnicity data retrieved from healthcare datasets, is the accuracy of recorded ethnicity data. Previous studies have demonstrated imperfect agreement between ethnicity data in healthcare datasets and census.^24^ The ethnicity proportions in this study were similar to the ethnicity proportions in the 2021 Census of the area contributing the largest proportion of results. However, we could not assess the accuracy of ethnicity data or the completeness of recording. Recording of accurate granular ethnicity data in healthcare dataset will improve the accuracy and applicability of such future studies. Despite this limitation, ethnicity-specific reference intervals could play an important role in improving equity and mitigating ethnicity-related misclassification bias until more promising alternatives, for example personalised reference intervals, are widely adopted.^25^

Data on CK levels in people of mixed ethnicity are limited. Notably, our study provides the first evidence that CK results in individuals of mixed ethnicity tend to align with the higher of their two parent ethnic groups. We recommend applying the reference interval for the ethnic background associated with higher CK levels when interpreting results from individuals of mixed ethnicity. This is relevant in increasingly ethnically integrated communities with rising rates of mixed- heritage individuals.

In the UK, ethnicity information is recorded in standardised categories (Ethnic Category Code 2001) and stored in primary care records and hospital patient administration systems. This information can be transmitted to the LIMS with electronic orders, provided the data fields are correctly mapped. Currently, however, not all laboratories routinely import patient ethnicity into the LIMS. If implemented, this would enable laboratories to apply ethnicity-specific reference ranges or add interpretative comments for relevant subpopulations.

There are some limitations to consider. Because the reference intervals were derived from retrospective laboratory data, we could not individually exclude instances of physiologically or pathologically elevated CK, for example, elevations due to acute illness, strenuous exercise, medication side-effects or myopathies. However, we minimised such effects by including results only from primary care and a single CK result per individual to avoid over-representation of patients with chronic diseases or repeated CK elevations. Furthermore, we used a robust algorithm least susceptible to outliers to mitigate the impact of inadvertently included pathological results from individuals with myopathies and other conditions causing CK elevations.^16^, ^22^ These measures should have mitigated bias, ensuring that the derived CK reference intervals largely reflect healthy baseline levels. It is to be noted, however, that the robustness of the reference intervals, particularly for smaller sex and ethnicity categories, could be improved by future studies employing larger datasets. Another limitation is inability to derive CK reference intervals for individuals of mixed ethnicities, children <13 years and age-group stratified sex- and ethnicity-specific reference intervals because of dataset size limitations. Paediatric sex-specific CK reference intervals are available from the Canadian Laboratory Initiative on Paediatric Reference Intervals (CALIPER).^26^ However, our data (Fig. 3) suggest that CK URL in children under the age of 13 years from Black ethnic backgrounds is likely to be 1.2–1.3 times higher compared to those of White ethnic backgrounds, and CK URL in children under the age of 13 years from Asian ethnic backgrounds is likely to be similar or marginally higher compared to those of White ethnic backgrounds. Finally, clinicians should be mindful of the age-related decrease when interpreting CK results in older people. However, considering that CK generally increases multi-fold in most muscle disorders and myopathies, the likelihood of missed diagnoses by employing a single CK reference interval for adults will be small.^27^, ^28^

In summary, we provide first ever sex- and ethnicity-specific CK reference intervals for the UK population. We verify that adoption of these reference intervals will facilitate equitable clinical decision-making and improve patient care by minimising misdiagnosis and subsequent unnecessary investigations and treatment modifications. We demonstrate age-related changes in CK and sex- and ethnicity-specific differences in age-related changes. For the first time, we provide data to support that CK results in individuals of mixed ancestry align more with the higher of their two parent ethnic groups.

## CRediT authorship contribution statement

**Catherine Collingwood:** Writing – review & editing, Conceptualization. **Hem Sapkota:** Writing – review & editing, Conceptualization. **Emma Tuddenham:** Writing – review & editing, Data curation, Conceptualization. **Rachel L. Griffiths:** Writing – review & editing, Writing – original draft, Project administration, Methodology, Investigation, Formal analysis, Data curation, Conceptualization. **Latika Gupta:** Writing – review & editing, Conceptualization. **Mariam El Hawli:** Writing – review & editing, Conceptualization. **Tejas Kalaria:** Writing – review & editing, Writing – original draft, Supervision, Project administration, Methodology, Formal analysis, Conceptualization. **Rousseau Gama:** Writing – review & editing, Conceptualization.

## Ethics approval and consent to participate

Registered service improvement audits at institutions that contributed anonymised data that originated form routine care (Audit ID 17480 with the Royal Wolverhampton NHS Trust/ Black Country Pathology Services, and CBS-Audit-639 with South West London Pathology). Requirement for NHS Research Ethics Committee (REC) review was assessed using the UK Health Research Authority ʻDo I need NHS REC review?’ decision tool. The tool indicated that NHS REC review was not required for this study in England. The data were handled in compliance with GDPR and local information governance protocols.

## Funding

This research did not receive any specific grant from funding agencies in the public, commercial or not-for-profit sectors.

## Declaration of competing interest

The authors declare that they have no known competing financial interests or personal relationships that could have appeared to influence the work reported in this paper.

## Data Availability

The data that support the findings of this study are available from the corresponding author upon reasonable request. The datasets used for this study are not shared.

## Footnotes

Appendix A Supplementary data associated with this article can be found in the online version at doi:10.1016/j.clinme.2026.100596.

## References

[1] Neal R.C., Ferdinand K.C., Ycas J. (2009). Relationship of ethnic origin, gender, and age to blood creatine kinase levels. Am J Med.

[2] George M.D., McGill N.-K., Baker J.F. (2016). Creatine kinase in the U.S. population: impact of demographics, comorbidities, and body composition on the normal range. Medicine.

[3] Meltzer H.Y. (1971). Factors affecting serum creatine phosphokinase levels in the general population: the role of race, activity and age. Clin Chim Acta.

[4] Bright M., Wagman E., Shastri S. (1980). Race-related differences in reference intervals for creatine kinase. Clin Chem.

[5] Wong E.T., Cobb C., Umehara M.K. (1983). Heterogeneity of serum creatine kinase activity among racial and gender groups of the population. Am J Clin Pathol.

[6] Berg J., Lane V. (2011). Pathology harmony; a pragmatic and scientific approach to unfounded variation in the clinical laboratory. Ann Clin Biochem.

[7] Franck P.F., Steen G., Lombarts A.J. (1998). Multicenter harmonization of common enzyme results by fresh patient-pool sera. Clin Chem.

[8] National Institute for Clinical Excellence (2023). NICE Guidelines CG238.

[9] Mach F., Baigent C., Catapano A.L. (2020). 2019 ESC/EAS guidelines for the management of dyslipidaemias: lipid modification to reduce cardiovascular risk. Eur Heart J.

[10] Grundy S.M., Stone N.J., Bailey A.L. (2019). 2018 AHA/ACC/AACVPR/AAPA/ABC/ACPM/ADA/AGS/APhA/ASPC/NLA/PCNA guideline on the management of blood cholesterol: executive summary: a report of the American College of Cardiology/American Heart Association Task Force on clinical practice guidelines. J Am Coll Cardiol.

[11] NHS England. Ethnicity – NHS data dictionary (Ethnic Category Code 2001). 〈https://datadictionary.nhs.uk/attributes/ethnic_category_code_2001.html〉 Accessed 4 February 2025.

[12] Schumann G., Bonora R., Ceriotti F. (2002). IFCC primary reference procedures for the measurement of catalytic activity concentrations of enzymes at 37 °C. Part 2. Reference procedure for the measurement of catalytic concentration of creatine kinase. Clin Chem Lab Med.

[13] Aloisio E., Frusciante E., Pasqualetti S. (2020). Traceability validation of six enzyme measurements on the Abbott Alinity c analytical system. Clin Chem Lab Med.

[14] Eilers P.H.C., MB D., Maria D. (2015). Twenty years of P-splines. Stat Oper Res Trans.

[15] Rigby R.A., Stasinopoulos D.M. (2005). Generalized additive models for location, scale and shape. J R Stat Soc Ser C (Appl Stat).

[16] Ammer T., Schützenmeister A., Prokosch H.-U. (2021). refineR: a novel algorithm for reference interval estimation from real-world data. Sci Rep.

[17] Ammer T., Schützenmeister A., Rank C.M. (2023). Estimation of reference intervals from routine data using the refineR algorithm-A practical guide. J Appl Lab Med.

[18] How life has changed in Sandwell: Census; 2021. 〈https://www.ons.gov.uk/visualisations/censusareachanges/E08000028/〉 Accessed 6 January 2026.

[19] Brewster L.M., Mairuhu G., Sturk A. (2007). Distribution of creatine kinase in the general population: implications for statin therapy. Am Heart J.

[20] Jones G.R.D., Haeckel R., Loh T.P. (2018). Indirect methods for reference interval determination - review and recommendations. Clin Chem Lab Med.

[21] Kalaria T., Lawson A.J., Duffy J. (2024). Age-specific reference intervals of Abbott intact PTH-potential impacts on clinical care. J Endocr Soc.

[22] Ammer T., Schützenmeister A., Prokosch H.-U., Zierk J., Rank C.M., Rauh M. (2022). RIbench: a proposed benchmark for the standardized evaluation of indirect methods for reference interval estimation. Clin Chem.

[23] Farrell C.-J.L., Nguyen L. (2019). Indirect reference intervals: harnessing the power of stored laboratory data. Clin Biochem Rev.

[24] Razieh C., Powell B., Drummond R. (2025). Understanding the quality of ethnicity data recorded in health-related administrative data sources compared with Census 2021 in England. PLoS Med.

[25] More A.K., Lorde N., Kalaria T. (2025). Extended roles in healthcare delivery: what is the role of the laboratory in addressing ethnicity-related healthcare disparities?. Diagnostics.

[26] Tahmasebi H., Higgins V., Woroch A., Asgari S., Adeli K. (2019). Pediatric reference intervals for clinical chemistry assays on Siemens ADVIA XPT/1800 and Dimension EXL in the CALIPER cohort of healthy children and adolescents. Clin Chim Acta.

[27] Kyriakides T., Angelini C., Schaefer J. (2010). EFNS guidelines on the diagnostic approach to pauci- or asymptomatic hyperCKemia. Eur J Neurol.

[28] Moghadam-Kia S., Oddis C.V., Aggarwal R. (2016). Approach to asymptomatic creatine kinase elevation. Clevel Clin J Med.

