# Supplementary material for: Age, sex and ethnicity changes in creatine kinase and sex- and ethnicity-specific reference intervals of creatine kinase
Source: Clin Med (Lond). 2026 May 15;26(4):100596. doi: 10.1016/j.clinme.2026.100596 (PMC13263756; doi:10.1016/j.clinme.2026.100596)
Supplement: Supplementary file 1 — Supplementary material [file mmc1.pdf]

An example of commands in R Studio for derivation of reference intervals using package refineR

Key:

Dataset = Name of the data file loaded in R studio

CK = header name of column containing numeric CK results

ETHNIC_GRP2_ABW = Header name of the column containing ethnicity classifications

SEX = Header name of the column containing sex information

AGE_YEARS = Header name of the column with numeric age data in years

```
library(refineR)

library(dplyr)

# Filtering data to select Asian female age >=13 years

filtered_data <- Dataset %>% filter(ETHNIC_GRP2_ABW == "A", SEX == "F", AGE_YEARS >= 13)


# CK reference intervals for Asian Females Aged 13 Years and Older

resRIAF <- findRI(Data = filtered_data$CK, NBootstrap = 1000)

print(resRIAF)

getRI(resRIAF, RIperc = c(0.025, 0.5, 0.975), CIprop = 0.95, pointEst ="medianBS")

plot(resRIAF, RIperc = c(0.025, 0.500, 0.975), CIprop = 0.95, showPathol = TRUE, pointEst = "medianBS", xlab = "CK Level (IU/L)", ylab = "Frequency", title = "CK Reference Interval for Asian Females Aged 13 Years and Older")

plot(resRIAF, RIperc = c(0.025, 0.500, 0.975), CIprop = 0.95, showPathol = TRUE, pointEst = "medianBS", xlab = "Box-Cox Transformed CK Level", ylab = "Frequency", title = "CK Reference Interval for Asian Females Aged 13 Years and Older", Scale = "transformed")
```

Example R code in R Studio for plotting sex- and ethnicity specific median plots with P-splines using singular value decomposition function (pb()) in Box-Cox transformed distribution in GAMLSS

```
library(gamlss)

library(ggplot2)
```

# Fitting a separate GAMLSS model for each of six sex and ethnicity combinations using Box-Cox transformed BCCG distribution using pb(). The below example is for Asian Females and assumes that prefiltered dataset of Asian Females called "asian_female" containing at least CK results and AGE_YEARS (age in years) is available.

```
fit_AF <- gamlss( CK ~ pb(AGE_YEARS), data = asian_female, family = BCCG)
```

# Predict the median curve (mu) across an age grid store as data frame

```
age_grid <- data.frame(AGE_YEARS = seq(0, 90, by = 1))
pred_AF <- age_grid
pred_AF$CK_median <- predict(fit_AF, newdata = age_grid, what = "mu", type = "response")
pred_AF$EthnicGroup <- "Asian"
pred_AF$Sex <- "Female"
```

# Repeat the above process for the other five sex- and ethnicity-groups to create pred_AM, pred_BF, pred_BM, pred_WF, pred_WM respectively for Asian Male, Black Female, Black Male, White Female and White Male.

# Final plot combining medians from all six groups' prediction tables

```
pred_all <- rbind(pred_AF, pred_AM, pred_BF, pred_BM, pred_WF, pred_WM)
pred_all$EthnicGroup <- factor(pred_all$EthnicGroup, levels = c("Asian", "Black", "White"))
pred_all$Sex <- factor(pred_all$Sex, levels = c("Female", "Male"))
ggplot(pred_all, aes(x = AGE_YEARS, y = CK_median, colour = EthnicGroup, linetype = Sex)) +
 geom_line(linewidth = 1) +
 scale_color_manual(values = c(Asian = "red", Black = "black", White = "blue")) +
 scale_linetype_manual(values = c(Female = "dashed", Male = "solid")) +
 labs(title = "Median CK Levels (IU/L) by Age, Sex, and Ethnic Group",  x = "Age (Years)",  y = "CK
Levels (IU/L)", colour = "EthnicGroup", linetype = "Sex") +
 coord_cartesian(xlim = c(0, 90)) +
 theme_minimal(base_size = 12) +
 theme(legend.position = "right")
```

Supplementary Figure 1: Distribution of CK levels by age in sex (F = female, M = male) and ethnicity (W = White, A = Asian, B = Black) groups.

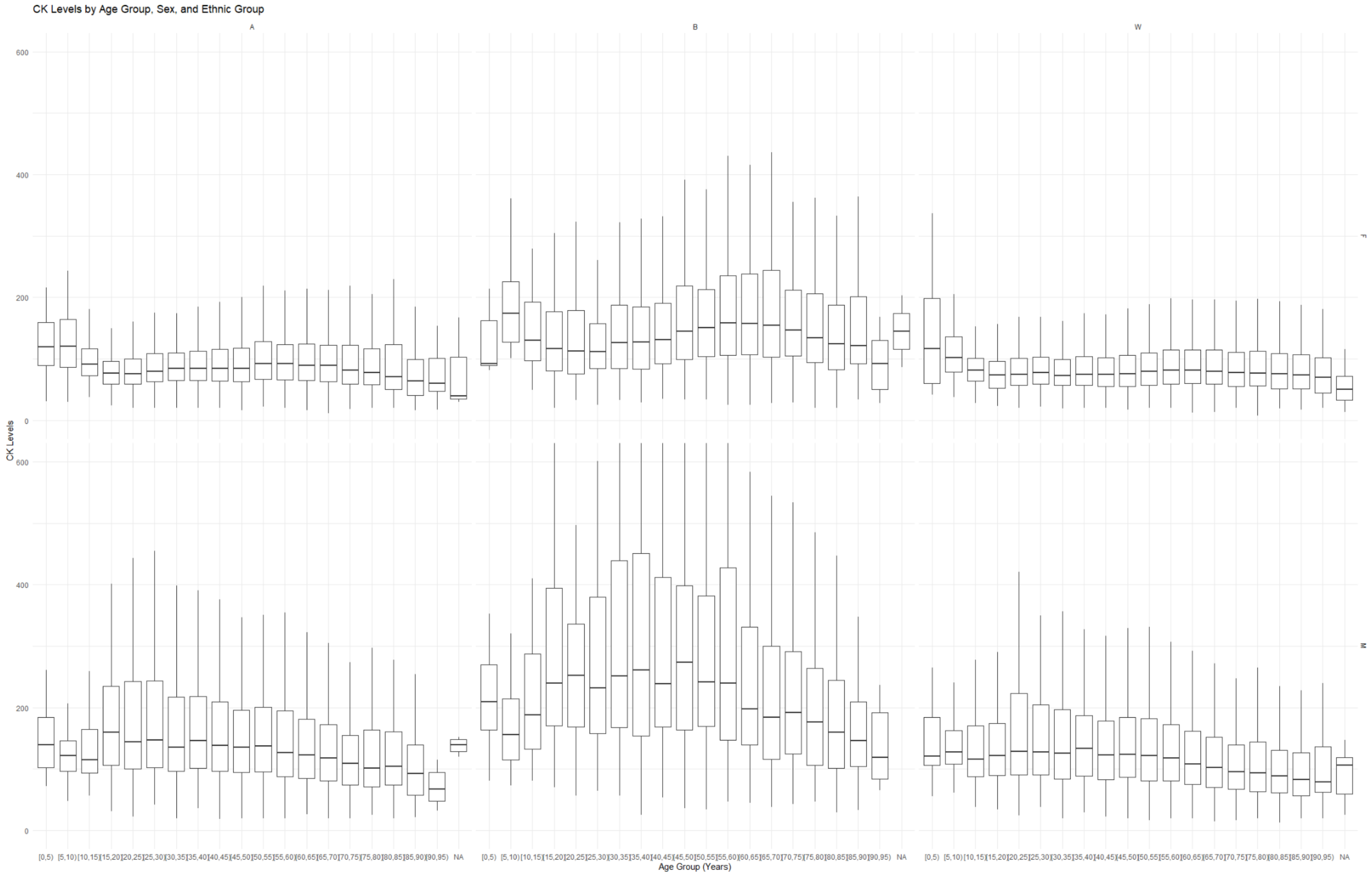

Supplementary Figure 2: Native and Box-Cox transformed CK distributions from refineR algorithm for sex and ethnicity groups. In order of increasing CK, the vertical dotted lines indicate 2.5th, 50th and 97.5th percentiles and the shaded regions encompassing the lines indicate 95% confidence intervals of the percentiles. The optimal distribution of non-pathological results identified by the refineR algorithm is indicated by a solid green distribution. The red line closer to the abscissa indicates results that were not part of the identified optimal non-pathological distribution and therefore excluded from percentile derivation.

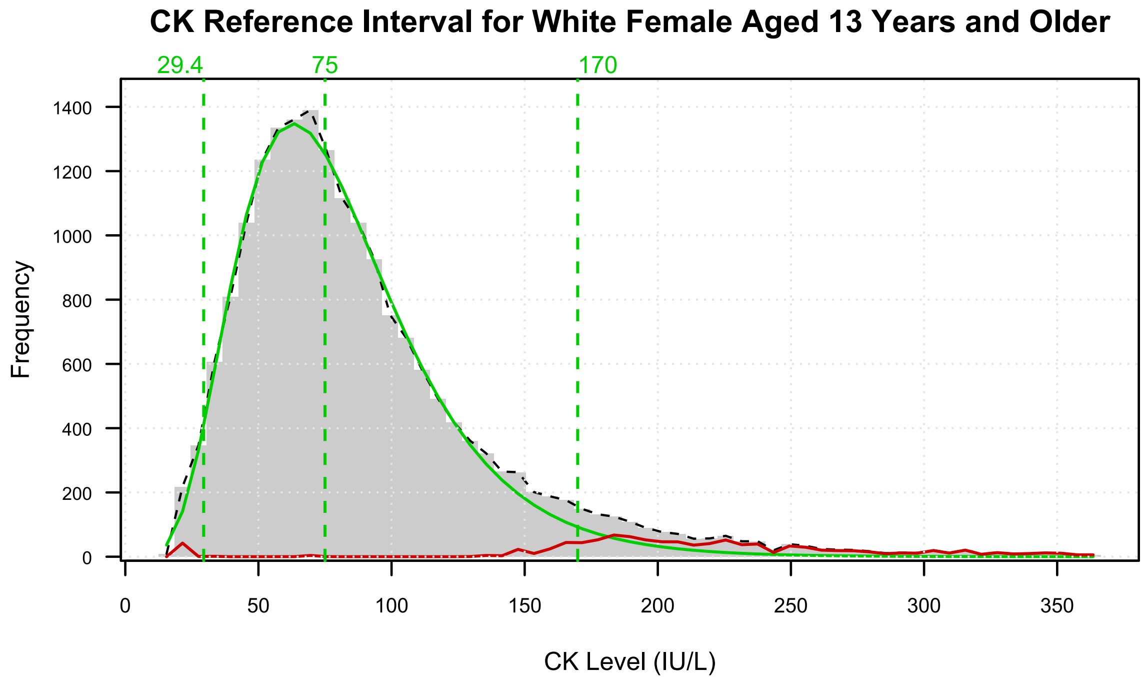

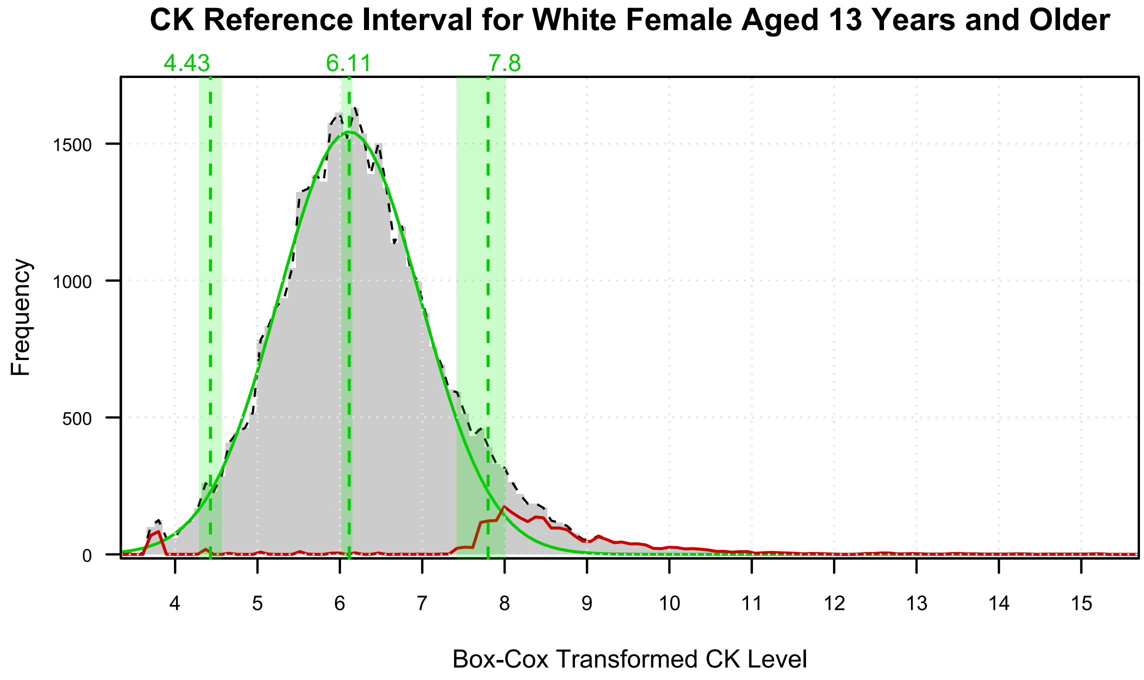

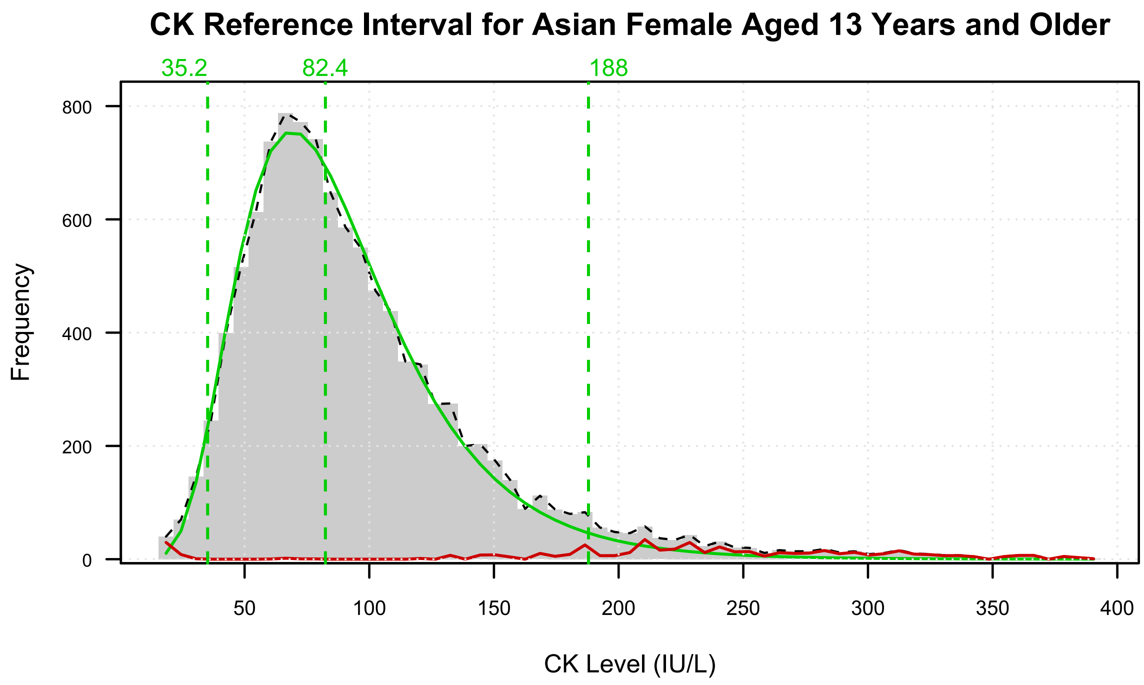
CK Reference Interval for Asian Female Aged 13 Years and Older
35.2
82.4
188
Frequency
0
200
400
600
800
50
100
150
200
250
300
350
400
CK Level (IU/L)

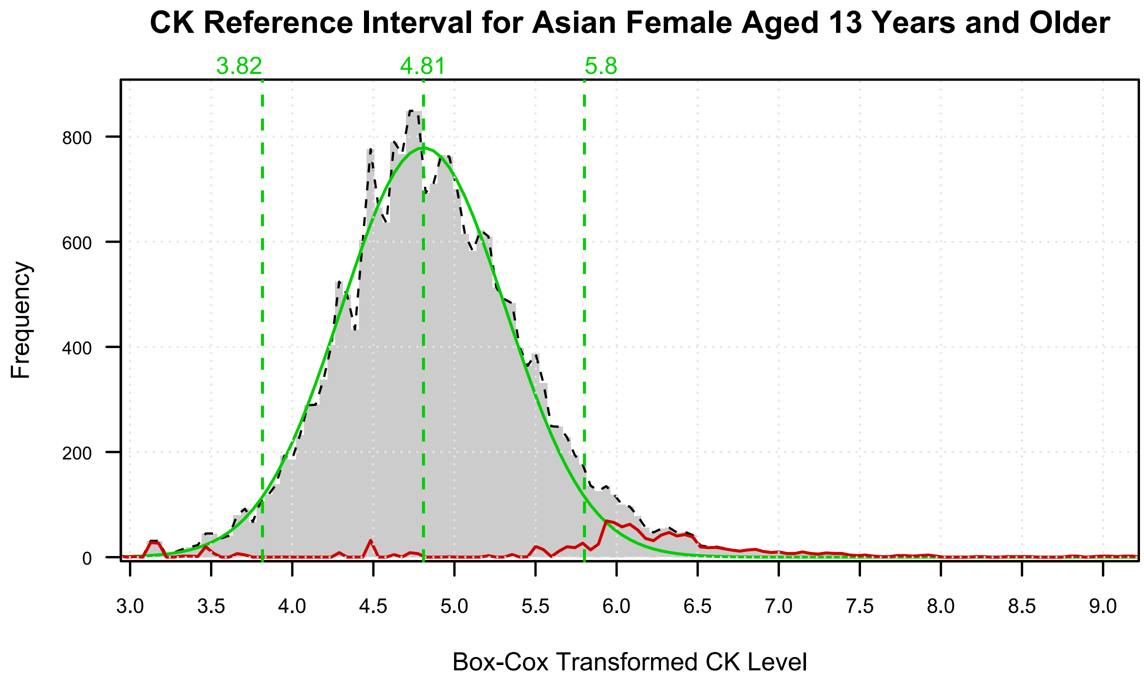
CK Reference Interval for Asian Female Aged 13 Years and Older
3.82
4.81
5.8
Frequency
0
200
400
600
800
3.0
3.5
4.0
4.5
5.0
5.5
6.0
6.5
7.0
7.5
8.0
8.5
9.0
Box-Cox Transformed CK Level

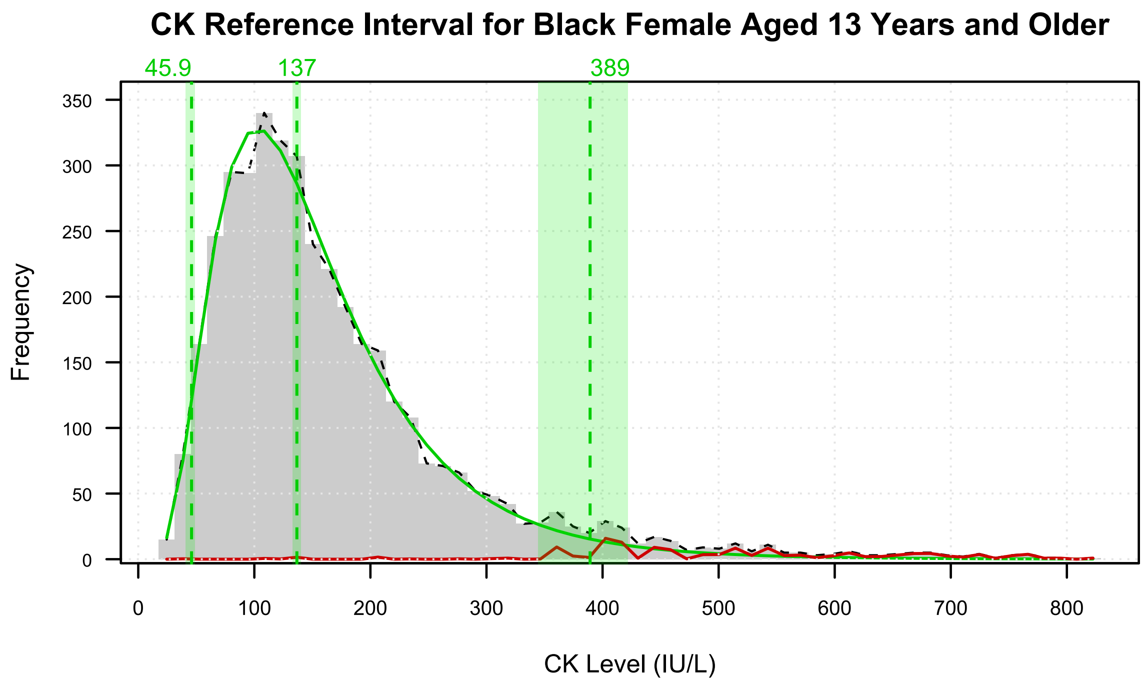

CK Reference Interval for Black Female Aged 13 Years and Older
45.9
137
389
Frequency
CK Level (IU/L)

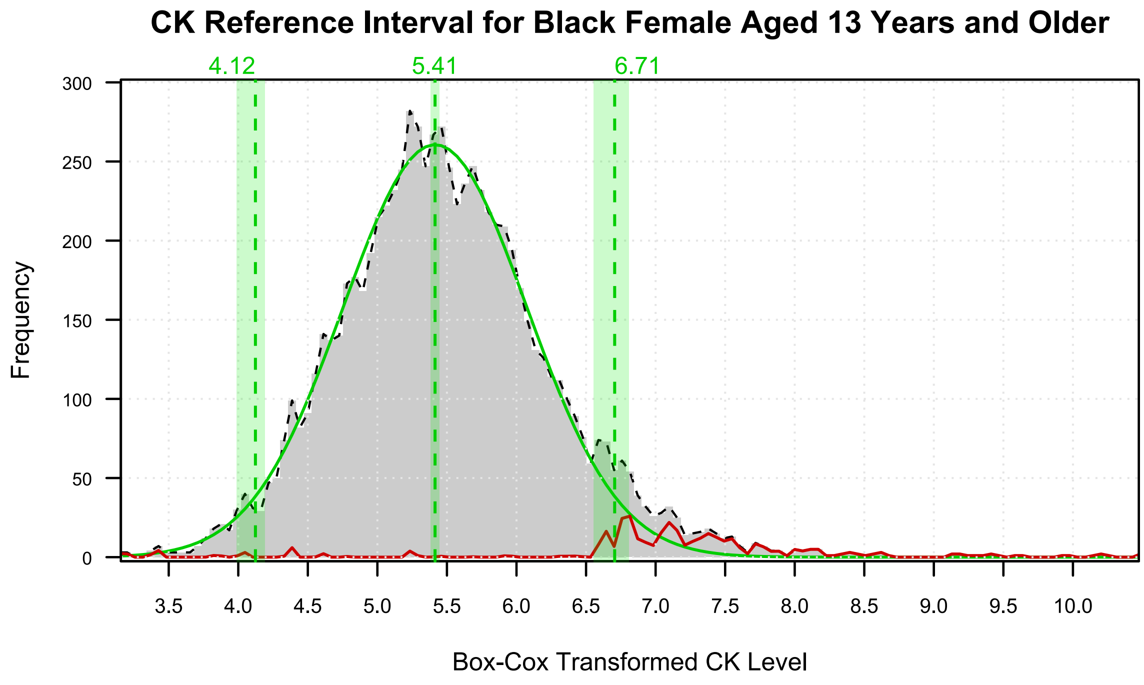

CK Reference Interval for Black Female Aged 13 Years and Older
4.12
5.41
6.71
Frequency
Box-Cox Transformed CK Level

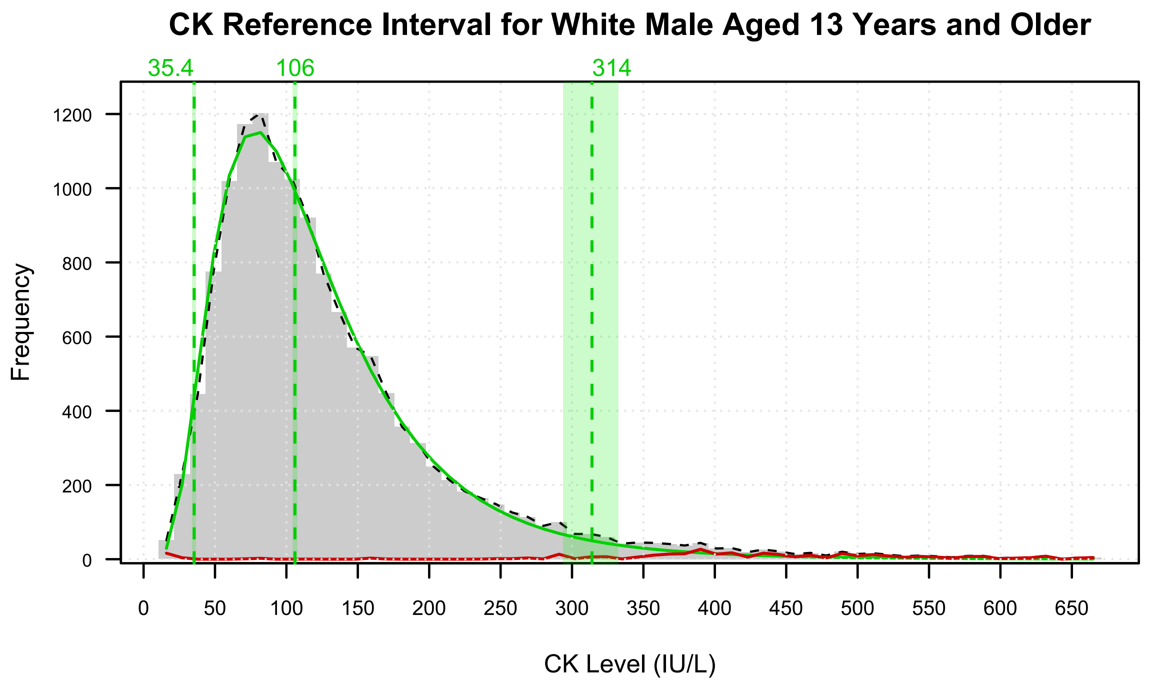
CK Reference Interval for White Male Aged 13 Years and Older
35.4
106
314
Frequency
0
200
400
600
800
1000
1200
0
50
100
150
200
250
300
350
400
450
500
550
600
650
CK Level (IU/L)

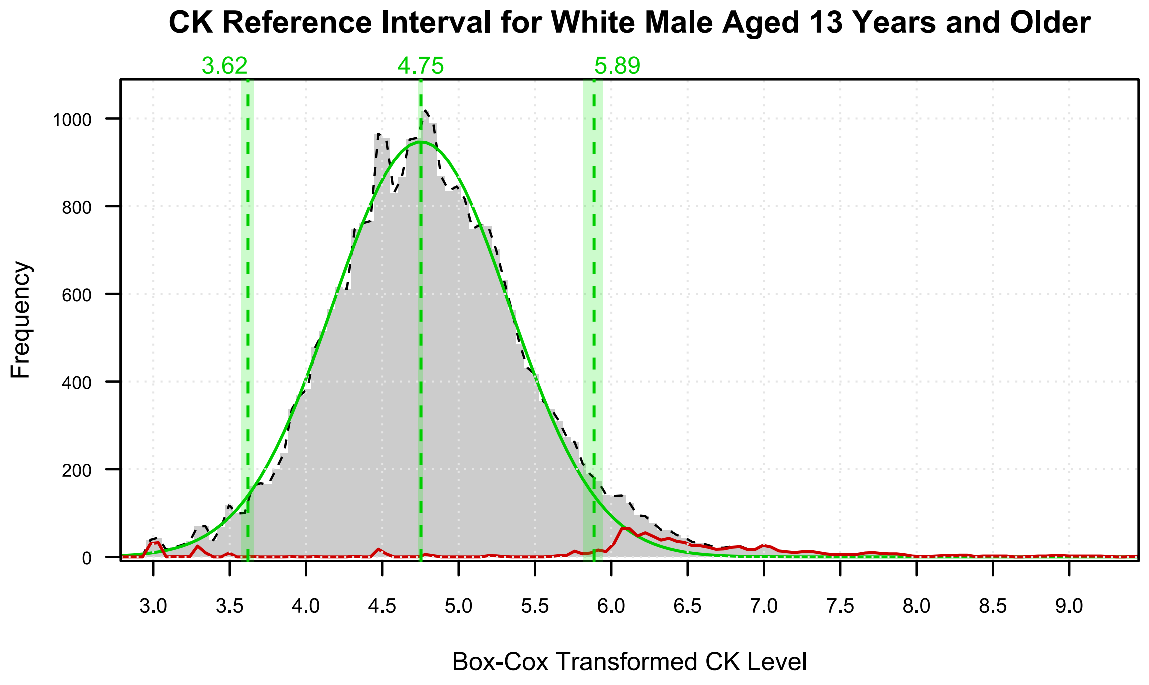
CK Reference Interval for White Male Aged 13 Years and Older
3.62
4.75
5.89
Frequency
0
200
400
600
800
1000
3.0
3.5
4.0
4.5
5.0
5.5
6.0
6.5
7.0
7.5
8.0
8.5
9.0
Box-Cox Transformed CK Level

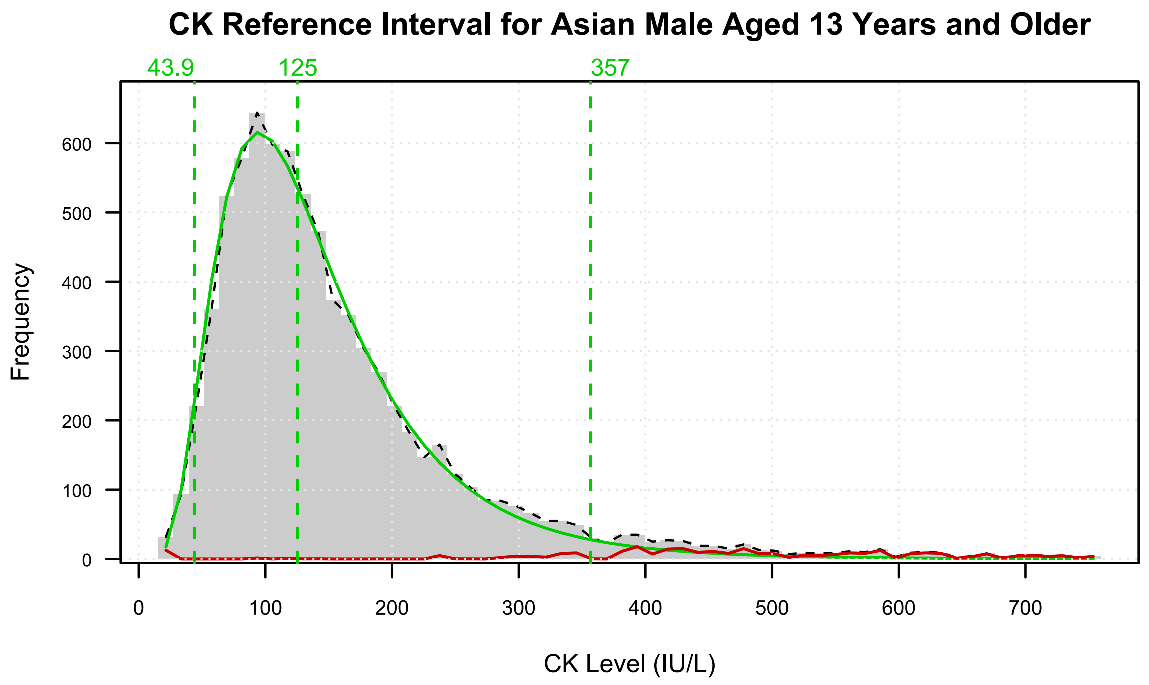
CK Reference Interval for Asian Male Aged 13 Years and Older
43.9
125
357
Frequency
0
100
200
300
400
500
600
700
CK Level (IU/L)

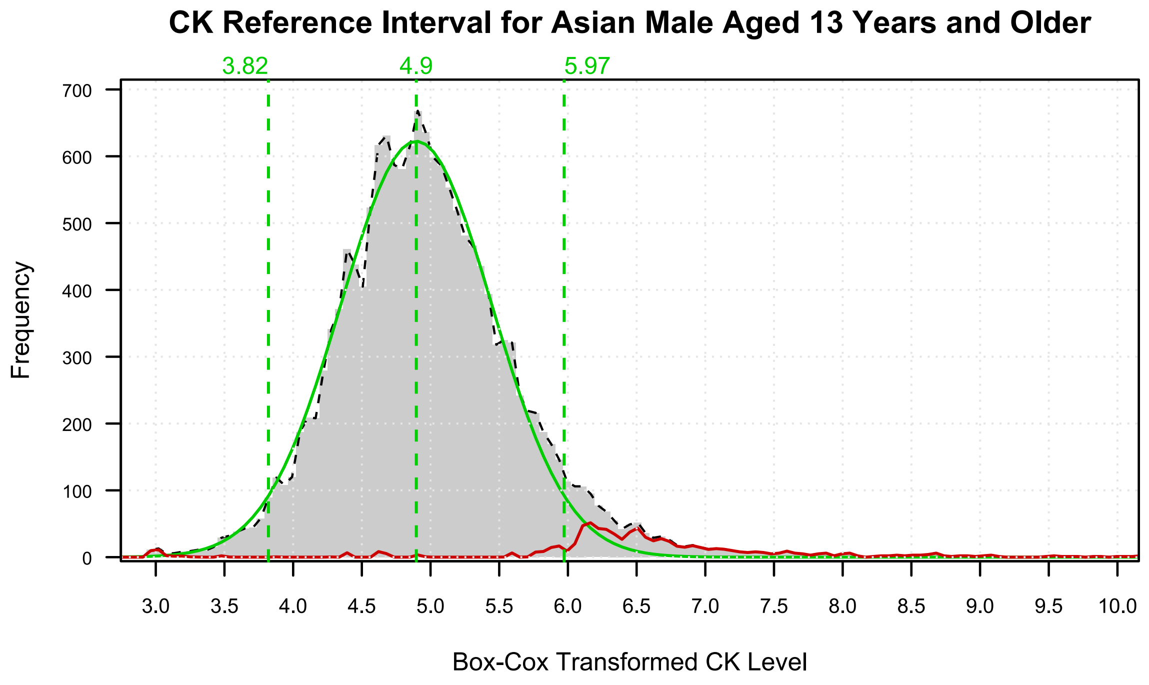
CK Reference Interval for Asian Male Aged 13 Years and Older
3.82
4.9
5.97
Frequency
0
100
200
300
400
500
600
700
3.0
3.5
4.0
4.5
5.0
5.5
6.0
6.5
7.0
7.5
8.0
8.5
9.0
9.5
10.0
Box-Cox Transformed CK Level

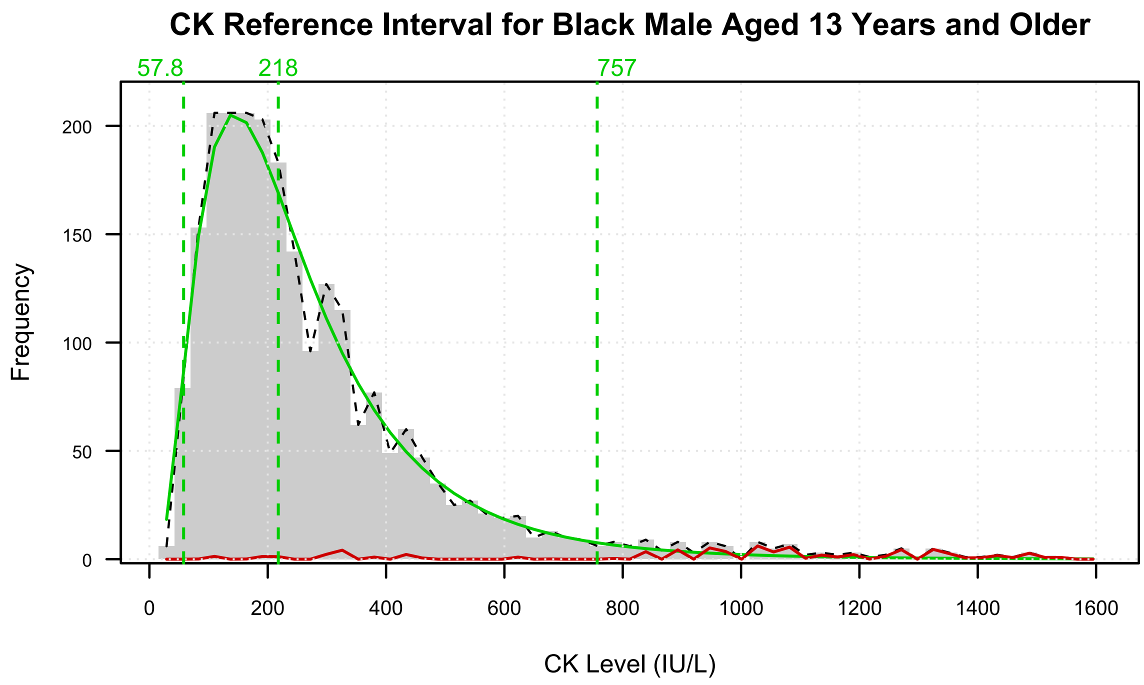
CK Reference Interval for Black Male Aged 13 Years and Older
57.8
218
757
Frequency
0
50
100
150
200
0
200
400
600
800
1000
1200
1400
1600
CK Level (IU/L)

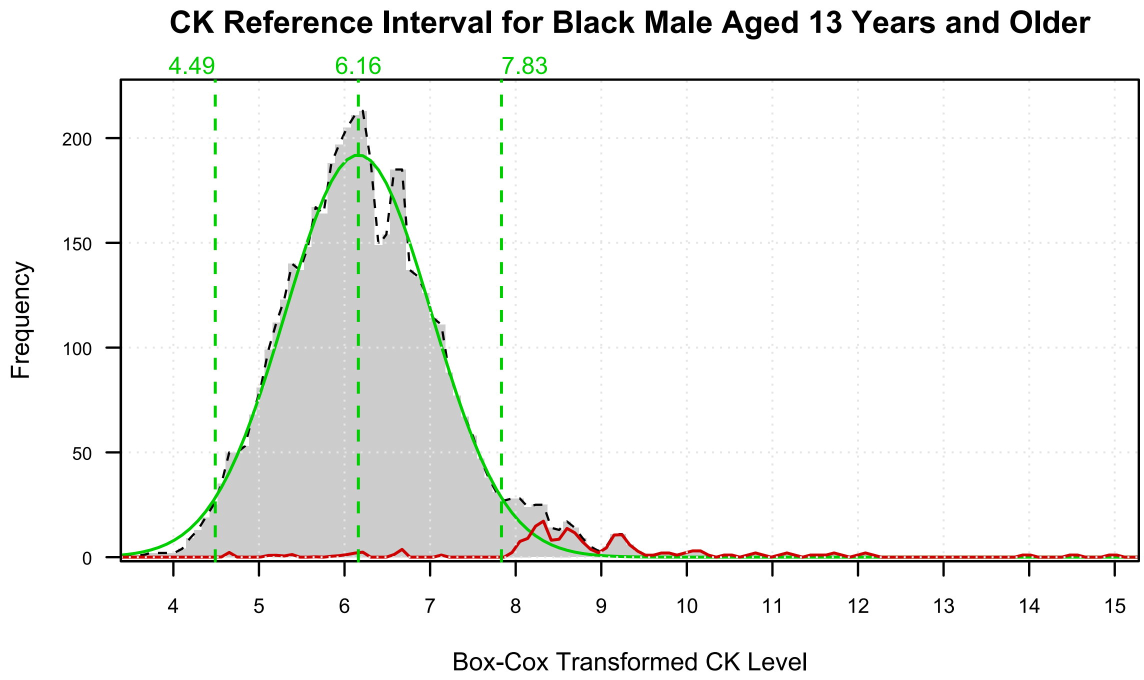
CK Reference Interval for Black Male Aged 13 Years and Older
4.49
6.16
7.83
Frequency
0
50
100
150
200
4
5
6
7
8
9
10
11
12
13
14
15
Box-Cox Transformed CK Level
